# The Insane in Texas

**Published:** 1913-02

**Authors:** 


### The Insane in Texas.

The following data I abstract from the annual report of the superintendent and board of management of the Southwestern Insane Asylum, San Antonio, Texas, for the year ending August 31, 1912.

Dr. White, the superintendent, says:

In order that a more comprehensive idea may be formed of the conditions existing in Texas at this time, it is deemed proper to give a short résumé of the provision for and the care and treatment of the insane in Texas, from the establishment of the first asylum until the present time.

The State Lunatic Asylum was established in Austin in 1857, and in 1860 it contained only 60 patients. In the attempt to meet the growing demands of a rapidly developing State, this institution has been extended and added to until today it accommodates 1600 patients, and the improvements, including land, are valued at $600,000.

As the population of the State grew north and west the demands for more accommodations were met by the establishment of the North Texas Hospital for the Insane at Terrell, which was ready for occupancy in July, 1885, and had a capacity of 400 patients. It has been found necessary to enlarge this asylum until today it accommodates 2225 patients, and represents a money value of $900,000.

The increase in population and the increase of insanity continuing called for more room, which was provided by the establishment of the Southwestern Insane Asylum, at San Antonio, which was opened in April, 1892, with a capacity of 200 patients. Today it provides for 1140 cases, and has a property value of $500,000.

In addition to these institutions for the insane, a colony for epileptics has been established at Abilene, which provides for 474 epileptics, and is valued at $350,000.

The combined number of inmates cared for in all of these institutions is 5439, and the combined valuation of the property is $2,350,000; in a little over a half a century these wards of the State have increased from 60 to 5439.

The following statement will present these facts in a more concise and impressive manner:

In 1860 the population of Texas was 604,251, and there were 60 patients in the State Lunatic Asylum, or 1 to 10,007 of population.

In 1870 the population was 818,579, with 83 inmates in the State Lunatic Asylum, or 1 to 9862 population.

In 1880 the population of the State had increased to 1,591,749, and the State Lunatic Asylum had 439 patients, or 1 to 3645 of the population.

In 1890 the population was 2,235,523, and there were in the

State Lunatic Asylum and the North Texas Hospital for the Insane 1113 patients, or 1 to 2008 of the population.

In 1900 the population of the State was 3,048,710, and the number confined in the State Lunatic Asylum, the North Texas Hospital for the Insane, and the Southwestern Insane Asylum was 2599, or 1 to 1171 of the population.

In 1910 the population of the State had increased to 3,896,542 and there were confined in the three lunatic asylums 4810 cases, or 1 to 810 of the population. There are today confined in the three insane asylums 4965.

In addition to this, there are 474 epileptics confined in the epileptic colony at Abilene.

A recent canvass of the State has been made by addressing a letter to each county judge, asking for certain data, which is contained in the following table, made up from reports of 223 counties. Twenty-two county judges failed to respond, but most of them are from the most sparsely populated sections of the State.

| | Males. | Females. | Total. |
|---|---|---|---|
| Number insane in county jails | 92 | 39 | 131 |
| Number insane on county farms | 39 | 35 | 74 |
| Number insane living with relatives | 93 | 67 | 160 |
| Total insane [not cared for by the State] | 224 | 141 | 365 |

Dr. White says further, under the heading

INCREASE AND PREVENTION OF INSANITY.

A very safe estimate of the relative importance of the different causes of insanity is given by Dr. Hubert Work, who says that: "Approximately 65 per cent of the insane are such through heredity; 15 per cent from alcoholism and drug addiction, the predisposition to which is from parents, not themselves necessarily inebriates, but of abnormal mentality; 10 per cent are from syphilis, inherited or acquired; the remaining 10 per cent from senility and other causes. Seventy-five per cent, in fact, will not cover all forms of intellectual defect due to heredity."

Our humane and improved methods of caring for the insane foster the increase of insanity. The mentally deranged are sent to a hospital for the insane, and through scientific care and treatment are soon restored to their normal condition and are sent out to propagate their species and bequeath to them an unstable organization, which will, under any extraordinary strain, break down and find a home in probably the same hospital that sheltered the parent.

This goes on from generation to generation, and we stand idly by and compliment the foresight and philanthropy of our times. There are two means which must be utilized to stop the increase of insanity, and these are education and surgery.

The people must be taught the evils which follow the marriage of those who are tainted with any form of disease. It should be a disgrace for any one to be the parent of a defective child which is the result of heredity.

A law should be passed requiring that all defectives be sterilized and thus prevented from transmitting to offspring their own unstable organization.

A very simple and safe surgical operation accomplishes this, and does so without unsexing the patient. Already the States of Iowa, Indiana, Oregon, Utah, Connecticut, and California [New York and New Jersey] have such laws. Sterilization is the procedure that goes right to the origin of the evil, and every State should legalize it by laws of liberal scope. This question should receive the earnest consideration of the Legislature.

Limit of space prevents a fuller extract from this splendid and exhaustive report. His remarks on the subject of prevention are especially timely, as we have now before the Texas Legislature a bill to sterilize all the inmates of the asylum who should not be permitted to hand down to future generations their degenerate and worthless progeny.

